# Pattern of survival of breast cancer patients in a tertiary hospital in South West Nigeria

**DOI:** 10.3332/ecancer.2021.1192

**Published:** 2021-02-25

**Authors:** Musa Ali-Gombe, Muhammad Inuwa Mustapha, Ayorinde Folasire, Atara Ntekim, Oladapo Babatunde Campbell

**Affiliations:** 1 Department of Radiology, College of Medical Sciences, Gombe State University, P.M.B 127, Gombe, Nigeria; 2 Department of Radiology, Aminu Kano Teaching Hospital, P.M.B 3452, Kano, Nigeria; 3 Department of Radiation Oncology, College of Medical Sciences, University of Ibadan, P.M.B 3017, Ibadan, Nigeria

**Keywords:** breast cancer, survival, Nigeria

## Abstract

**Background of the study:**

Breast cancer is the most common cancer among women in both developed and developing nations. The survival of breast cancer is increasing in developed countries with improved treatment modalities, while still very poor in developing countries. In Nigeria, few breast cancer survival data are available.

**Research design:**

This is a retrospective cross-sectional study.

**Objectives:**

To determine the survival of breast cancer patients and possible factors influencing it.

**Methodology:**

Socio-demographic and clinical variables from treatment records and case notes of breast cancer patients treated from 1 January 2004 to 31 December 2008 at the Department of Radiation Oncology, University College Hospital, Ibadan. The status of patients was determined at 2 and 5 years after diagnosis. The survival of patients with breast cancer was compared using Log Rank test according to socio-demographic and clinical variables. The median survival times were obtained from the Kaplan–Meier survival curve. Cox’s proportional hazard model was fitted for those that were statistically significant in the Log Rank test. Missing data were reported as unknown, not documented or missing.

**Results:**

A total of 378 patients were analysed. Age ranged between 22.0 and 87.0 years with mean of 47.6 (standard deviation (SD) = 11.2) years. Almost all patients were females (98.4%). More than half (55.3%) presented at stage III, 28.0% had metastasis and the stage was unknown in about 6.6% of the patients. Invasive ductal carcinoma was the most prevalent histology (89.2%). Only 124 (32.8%) patients had their histological grade stated and most of the patients had no immunohistochemistry done. All the patients had radiotherapy, chemotherapy and surgery. About 25.1% of the patients were lost to follow up. The 2- and 5-year survival rates were 56.4% and 37.6%, respectively. The 2- and 5-year survival rates according to stage were stage I (80.0% and 66.7%), stage II (67.7% and 57.6%), stage III (51.4% and 27.9%) and stage IV (37.9% and 13.8%). Median survival time was 41 months (95%CI = 35.0–44.0). The disease-free survival at 2 and 5 years was 66.6% and 60.3%, respectively. Median time for recurrence was 8.0 months. Level of education, height, tumour unilaterality, clinical tumour size, stage at presentation, presence of distant metastases, clinical axillary lymph node metastasis, supraclavicular node metastasis, mode of surgery and axillary clearance were found to have statistically significant association with survival.

**Conclusion:**

A large number of the patients in our study presented at a young age, late with advanced stage disease which results in poor survival outcome.

## Introduction

Breast cancer is the most frequent cancer among women with an estimated 2,088,849 million new cancer cases diagnosed in 2018 (11.6% of all cancers in the world), accounting for almost 1 in 4 cancer cases among women [1]. It ranks second overall after lung cancer in terms of both incidence and mortality [1]. It is also the most common cause of cancer death in women with about 626,679 (6.6%) deaths reported in 2018 [1].

Breast cancer continues to remain one of the most deadly malignancies in women across the world. In West Africa, it is a leading cause of cancer and cancer deaths among women with about 33% of new cases and 24% of cancer deaths in 2018 [2]. However, the incidence is significantly lower in Eastern Africa with approximately 20% of all new cancer cases and 15% deaths during the same period [2]. Incidence rates vary nearly fourfold across the world regions, with incidence from 27 per 100,000 in Middle Africa and Eastern Asia to 96 in Western Europe [3]. The highest incidence is seen in Australia/New Zealand at 94.2 per 100,000 [1].

Data from the Ibadan Cancer Registry put breast cancer as the commonest female malignancy comprising 40.8% of all cancers [4]. Studies have shown that age standardised incidence rates for breast cancer in Nigeria from 1960 to 2000 have more or less doubled over four decades with approximately 25% increase per decade [5]. A review of 5,000 cancer cases at the Radiotherapy Centre, University College Hospital (UCH), in Ibadan, Nigeria revealed that breast cancer was the commonest malignancy consisting of about 23% [6].

African women are diagnosed most often between 35 and 45 years, which is more than 15 years earlier than the women in Europe and North America with median age at diagnosis of 61 years [7].

Most patients in Nigeria present late; a study done in Lagos revealed that 66% of breast cancer patients who presented at the Lagos University Teaching Hospital were in stages III and IV disease whereby only palliation can be offered with eventual poor outcome [8]. In another study done at the Radiation Oncology Department, UCH, Ibadan, it was reported that 58.2% of the breast cancer patients presented late with advanced stage disease [9].

The 5-year overall survival rate varies from country to country, for instance, it is 88% in Canada, 90% in the united states [10], 80% and 64% in whites and blacks south Africans, respectively [11]. A study in Nigeria by Popoola *et al* [12] found that the overall 5-year survival in a population of breast cancer patients studied in Lagos was 25.6%.

The role of socio-demographic and clinical factors on survival of breast cancer patients has been explored by several authors worldwide with inconsistent findings.

There is a paucity of data on breast cancer survival studies in Nigeria. Most authors suggest that there is poor cancer survival in developing countries like ours largely due to disparity in cancer care across the world which includes ignorance, and lack of access to drugs, radiotherapy and expert care [13–15]. It is also considered that variation in race and tumour subtypes may also account for such differences [16]. Thus, it seems logical to consider the outcome and factors affecting it in a group of our patients with ‘optimal’ treatment. Hence, the need to study the pattern of survival of breast cancer patients at the Department of Radiation Oncology, UCH, Ibadan.

## Materials and methods

The study was carried out at the Department of Radiation Oncology, UCH, Ibadan, Nigeria. UCH is located in Ibadan North, Local Government Area of Oyo State, Nigeria. The clinic serves as a referral centre for oncology services in the South West, South East and South South regions of the country during the period under review.

All patients with histological diagnosis of breast cancer treated at the Radiation Oncology Department, UCH from 1 January 2004 to 31 December 2008 who met the study criteria were analysed.

### Inclusion criteria

- All histologically confirmed breast cancer patients (including ductal carcinoma in situ (DCIS)).
- Only patients that received optimal treatment were analysed. Patients were considered to have optimal treatment if she/he has completed the following:
  1. Breast conserving surgery (BCS) or mastectomy.
  2. Received at least four cycles of chemotherapy.
  3. Had at least 45 Gy to the breast/chest wall.

- Patient that had at least one follow up visit.
- Patients lost to follow up but with record of mobile phone number in the case note.

### Exclusion criteria

- Suspected breast cancer cases without histologic diagnosis or with conflicting histology.
- Cases with scanty treatment records. Cases with important missing data that will not allow for survival analysis such as date of diagnosis, date of death and date of recurrence.

### Censored data

Breast cancer patients were censored if

- The patient can no longer be observed (loss to follow up).
- The patient died of a disease quite different from breast cancer.
- Survival beyond the period of the study (5 years).

### Study design

This is a retrospective cross-sectional study.

## Data description and method of collection

All available radiotherapy case notes and treatment records of the breast cancer patients seen from 1 January 2004 to 31 December 2008 were retrieved. Information obtained from radiotherapy case notes and treatment records were patients’ bio-data including age at presentation, sex, patient’s and relative’s telephone numbers, ethnic group, occupation, marital status, employment and educational level. Socioeconomic level was classified into classes 1–6, clergy and retired according to Boroffka and Olatawura (1977) [17].

Duration of illness, site of disease and pathological features of the disease like clinical tumour size, stage at presentation, nodal status, histology, grade either as well differentiated (G1), moderately differentiated (G2) or poorly differentiated (G3), hormonal status of the tumour and the sites of metastases were also documented.

These data were determined from the referral letters, history, physical examination, histology reports, radiological and blood test during pretreatment evaluation, and follow up periods. The patients were retrospectively restaged at time of presentation using the 2010 edition of the American Joint Committee on Cancer. The details of treatment received was taken into consideration; radiotherapy (dose and duration), type of surgery (breast conserving or mastectomy), chemotherapy regimen, axillary clearance and hormonal therapy or combination of these.

The status of patients was determined at 2 and 5 years after diagnosis. The time of completion of treatment, time of first loco-regional recurrence and/or metastasis after completion of treatment were also noted. The status was either survival beyond end of study, loss to follow up or death. The time in months when the patient died or got lost to follow up or if they are still on follow up was recorded. The patients lost to follow up were contacted using their telephone numbers or that of their relatives which were recorded in the case note in order to ascertain their current status, and if they were dead, the date of death was obtained.

Two assistants with experience in data collection and fluent in English, Yoruba and Igbo were employed for this purpose as majority of patients were either Yoruba or Igbo. The patients who reside in Ibadan and could not be contacted through their phone numbers, were visited to ascertain their current status. The status of patients at 2 and 5 years after diagnosis was noted. The above information was extracted from records using a data extraction form. Missing data were reported as not documented, unknown or missing. However, if the missing data involve those in the inclusion criteria, the patients were excluded.

## Data management

The data were carefully entered, cleaned and analysed using STATA version 10.0. Regular cleaning and editing were done to detect and correct errors. Frequency tables were done for all the variables collected, and the sample size was compared with the number for each variable to ensure they coincided in number.

## Data analysis

Socio-demographic, clinical and treatment variables of patients were presented in tables, and pie charts using frequency, percentage, mean, median, range and SD.

Survival curves were calculated using the Kaplan–Meier method and curves were compared using Log rank test. Log rank test was used to test for an association between dependent variable (survival) and independent variables such as age, sex, marital status, educational status, employment status, social class, ethnic group, anthropometric parameters, duration of illness before presentation, stage of disease, clinical tumour size, metastasis, lymph node status, comorbidities, histology, grade, mode of surgery, axillary clearance and hormonal treatment. The analysis was considered to show significant association when the *p* value was ≤0.05.

The variables were recoded into groups for ease of comparison between the subgroups. The variables recoded were age; grouped into younger patients (≤40 years) and older patients (above 40 years), employment status; employed and unemployed, marital status; into married and not married (single, divorced, widow), social class; high (class 1–2), middle (class 3–4), low (class 5–6) and unclassified (clergy and retired), height (m); ˂1.5, 1.5–1.7, >1.7, weight (kg); ≤60, >60, Body mass index (BMI); normal weight, overweight and obese, duration of breast cancer symptoms; into ≤12 months and >12 months, clinical tumour size (cm); ˂2, 2–5, >5, Tumour, Node, Metastasis (TNM) stage; into early stage (stages I and II) and late or advanced stage (stages III and IV), axillary node status; into negative axillary nodes, positive ipsilateral axillary nodes and positive contralateral axillary nodes, histological grade; into well, moderate and poor differentiation and metastasis; into presence of metastasis and absence of metastasis. The treatment modalities were also grouped similarly; chemotherapy and radiotherapy (neo-adjuvant and adjuvant), surgery into breast conserving or mastectomy, whether the patient had axillary clearance or not and also if treated with hormonal therapy or not.

In the survival analysis using Kaplan–Meier, time of origin was the time of breast cancer diagnosis. The patient status as at 31 December 2013 was categorised as alive, dead and lost to follow-up. The endpoint of patient was death, patient loss to follow up or found alive by 31 December 2013 was censored. The median survival times were obtained from the Kaplan–Meier survival curve.

Multivariate analysis was carried out using Cox-proportional hazard models to determine the predictors of survival time. This was done using covariates to show statistically significant association with survival time at *p* ≤ 0.05 on bivariate analysis.

## Results

### Socio-demographic characteristics of respondents

A total of 378 out of an estimated 774 breast cancer patients over the period of study who met the selection criteria were analysed. Age of breast cancer patients ranged between 22.0 and 87.0 years with the mean age being 47.6 (SD = 11.2) years. The socio-demographic characteristics of the respondents are shown in Table 1.

### Physical characteristics of respondents

Physical characteristics of the patients are presented in Table 2. Height ranged 1.0–1.9 with a mean (SD) of 1.6 (0.1) m. Majority of the patients had heights between 1.5 and 1.7 m (70.1%). The weight ranged was 44.0–134.7 with a mean (SD) of 72.0 (14.2) and 198 (63.2%) had a weight greater than 60 kg. Eighty-six patients did not have height documented and 90 no weight in the records, so BMI was done for 90 patients. In the patients who had BMI calculated, about one third of the patients were overweight (31.0%).

The duration of illness ranged from 0–180 months with a mean of 18.4 (SD = 20.6) months. The majority of patients (78.8%) presented with first complain after 6 months. In 198 (52.4%) patients, the duration of illness was ≤12 months, while in 154 (40.7%) it was >12 months. In 26 patients (6.9%), there was no documentation of duration of illness in the history. There were 220 (58.2%) that were premenopausal and 128 (33.9%) post-menopausal, and in the remaining 24 women, menopausal status was not documented.

### Tumour characteristics and metastasis

**Figure 1.**
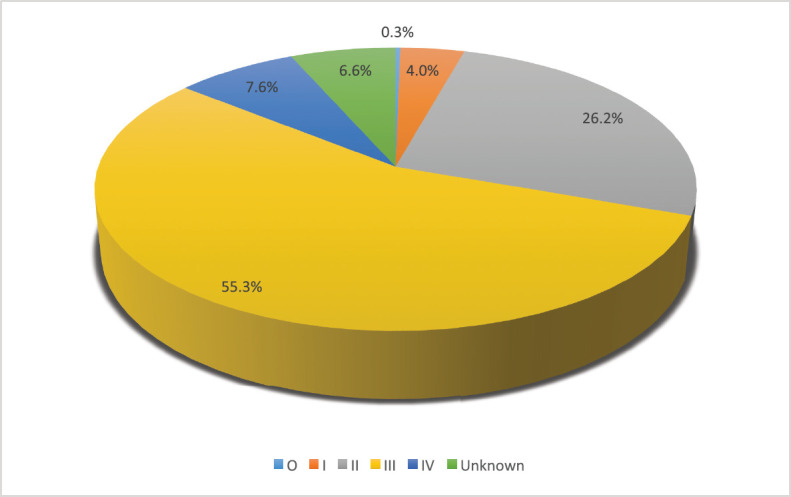
Distribution of patients’ stage at presentation.

Tumour characteristics and metastasis are shown in Table 3. About half of the patients had tumours on the left breast (50.3%). The mean clinical size of tumours was 7.3 (SD = 4.5) cm. (55.3%). Bone (9.5%), liver (6.1%) and lung metastasis (15.3%) were low. About 58.5% had palpable ipsilateral axillary lymph nodes metastasis. Only 31 patients (9.3%) had supraclavicular lymph nodes. Stage at presentation is shown in Figure 1 with about half presenting at stage III.

Figure 1 shows the distribution of patients’ stage at presentation, most of the patients (209) were in stage III. Only one patient presented at stage 0. In 25 (6.6%) of the patients, the stage was unknown.

### Histology

**Figure 2.**
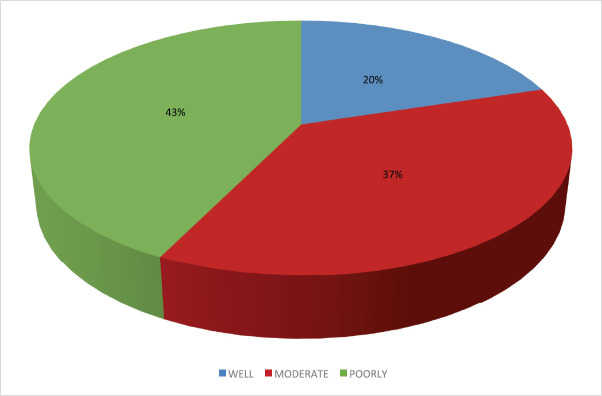
Distribution of SBR grade.

Histological profile of the patients is presented in Table 4. Invasive ductal carcinoma was the most prevalent (89.2%) followed by invasive lobular carcinoma (6.6%) and others (4.2%). The other histological types include medullary carcinoma (6), mucinous carcinoma (5), ductal carcinoma *in situ* (1), Paget’s disease (1), comedo carcinoma (1), stromal sarcoma (1) and squamous cell carcinoma (1). In the 124 who had Scarff–Bloom–Richardson (SBR) histological grading, poorly differentiated carcinomas were the majority; this is shown in Figure 2.

### Treatment modalities

Table 5 shows the distribution of treatment modalities of the patients. Majority received adjuvant chemotherapy (89.4%). A large number of the patients received Cyclophosphamide, Adriamycin, 5-FU (CAF) (42.3%). Adjuvant radiotherapy was received by 98.7% of patients. Most patients had mastectomy (86.8%) as opposed to BCS (13.2%). Axillary clearance and hormonal treatment were received by 67.2% and 63.5% of the patients, respectively.

### Overall survival rate/disease free survival

**Figure 3.**
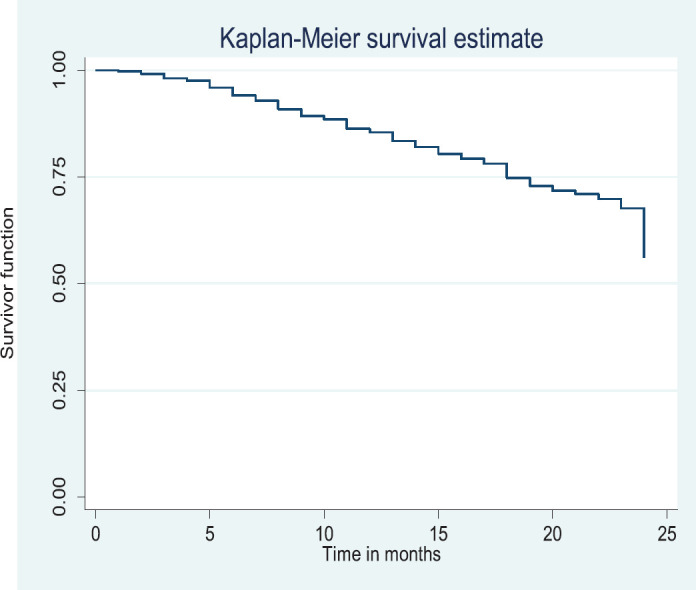
Two-year overall survival.

**Figure 4.**
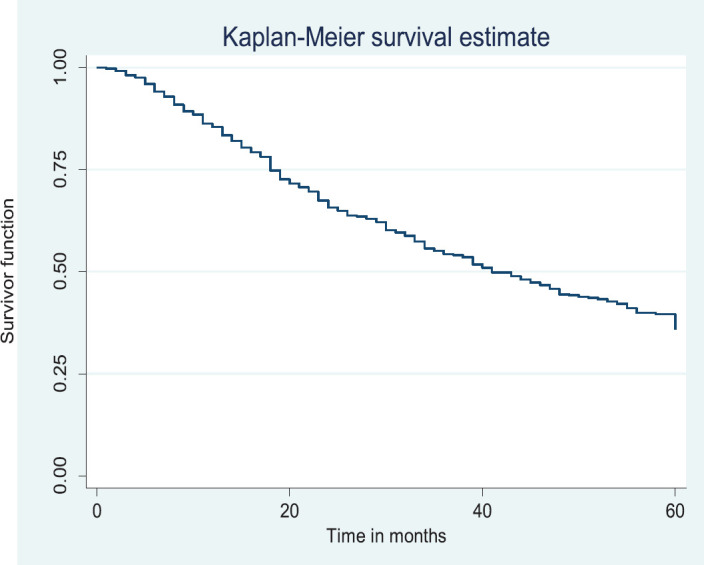
Five-year overall survival.

Out of the 378 patients that made the selection criteria, 25.1% were lost to follow up. The overall 2- and 5-year survival rates of the breast cancer patients studied were 56.1% and 37.6%, respectively. The overall 2- and 5-year Kaplan–Meier survival curves are shown in Figures 3 and 4. Median survival time for the whole population of patients was 41.0 months (95%CI = 35.0–48.0).

**Figure 5.**
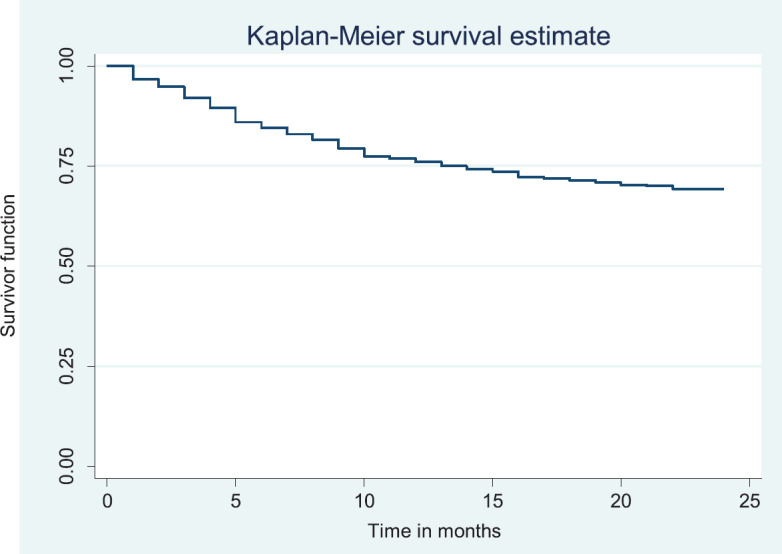
Two-year disease free survival.

**Figure 6.**
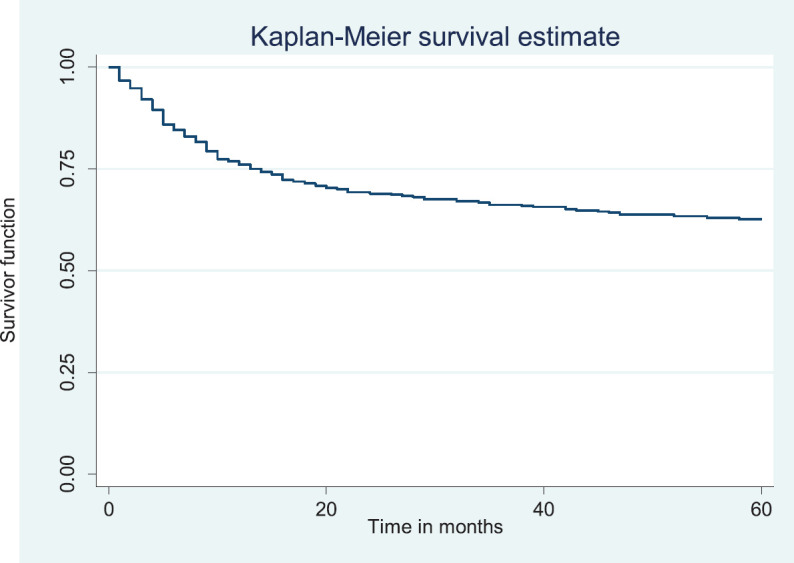
Five-year disease free survival.

The disease free survival at 2 and 5 years was 66.6% and 60.3%, respectively (Figures 5 and 6). Median time to recurrence was 8.0 months.

The above Kaplan–Meier curve depicts the 2-year overall survival (56.1%). Median survival time of the patients studied has not been reached at 2 years.

The above Kaplan–Meier curve shows the 5-year overall survival (37.6%). The median survival time was at 41.0 months (Figure 4) which was attained before 5 years (60 months).

The above Kaplan–Meier curve shows the 2-year disease free survival (66.6%). This shows that around two thirds of patients were alive at 2 years were free of disease.

The above Kaplan–Meier curve shows the 5-year disease free survival (60.3%). This shows that about 60% of the patients alive at 5 years were free of disease.

### Survival rates by selected variables

Table 6 shows the 5- and 2-year survival rates by selected variables.

### Comparative median survival times of patients among selected socio-demographic variables

Table 7 shows the comparative median survival times of patients among selected socio-demographic variables. Patients with formal education had a longer median survival time (48.0 months) compared to those with non-formal education (33.0 months), (*p* = 0.0309).

**Figure 7.**
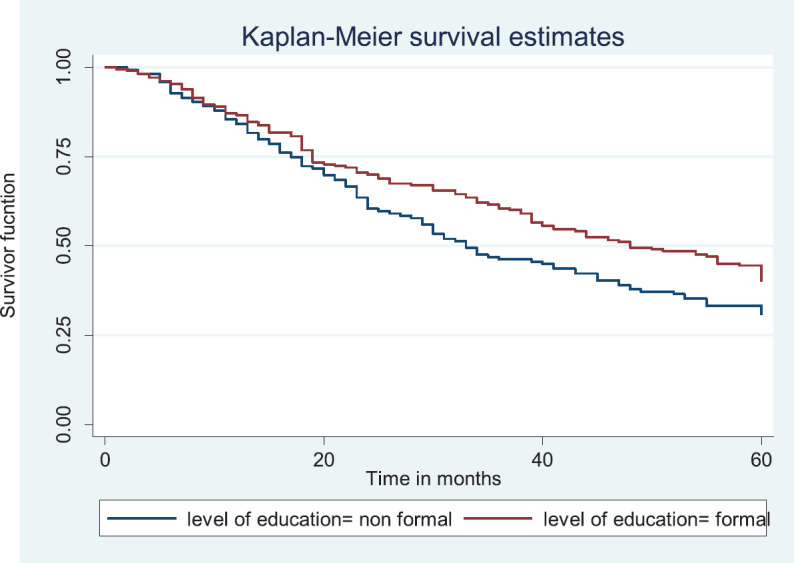
Kaplan–Meier curve by level of education.

Figure 7 depicts the Kaplan–Meier curve for level of education with median survival time of 48 and 33 months for formal and non-formal education, respectively. This was statistically significant Log rank = 4.66, *p*-value = 0.0309.

### Comparative median survival times of patients among anthropometric measurements and duration of illness

Comparative median survival times of patients among other selected variables are presented in Table 8. There was significant increase in median survival time with increase in height (*p* = 0.0153).

### Comparative median survival times of patients among tumour characteristics and metastases

Comparative median survival times of patients among tumour characteristics and metastases are presented in Table 9. Patients with bilateral tumours had a significantly lower median survival time (17.0 months) compared to those with right and left tumours (47.0 and 41.0 months), *p* = 0.0403. Also those with larger tumours >5 cm had a lower median survival of 38 months compared to those with tumours 2–5 cm whom median survival time had not been reached within the period of this study.

Patients with late stage of presentation had a significantly lower median survival time (36.0 months) compared to those with early stage at presentation whom median survival had not been reached at the period of the study, *p* < 0.001. Patients with liver metastasis had a significantly lower median survival time (20.0 months) compared to those without liver metastasis (45.0 months), *p* = 0.0015. Patients with lung metastasis had a significantly lower median survival time (30.0 months) compared to those without lung metastasis (47.0 months), *p* < 0.0005. Also patients with total metastasis (metastasis to the bone, spine, liver or cerebral) had a shorter median survival time (30.0 months) compared to those without metastasis (51.0 months), *p* < 0.001.

Similarly, patients with supraclavicular lymph node(s) had a shorter median survival time (24.0 months) compared to those who did not (41.0 months), *p* = 0.0033. Patients with non-palpable clinical axillary lymph nodes had a longer median survival time (43.0 months) compared to those with palpable ipsilateral (34.0 months) and palpable contra-lateral (23.0 months) axillary lymph nodes, *p* < 0.001.

**Figure 8.**
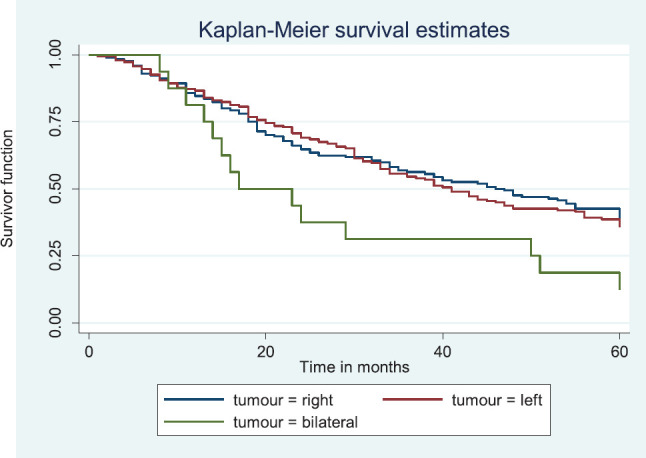
Kaplan–Meier curve by tumour site.

Figure 8 depicts the Kaplan–Meier curve for the tumour site with median survival time for unilateral (right; 47 months and left; 41 months) and bilateral (17 months) tumours. This was statistically significant Log rank = 6.42, *p*-value = 0.0403.

**Figure 9.**
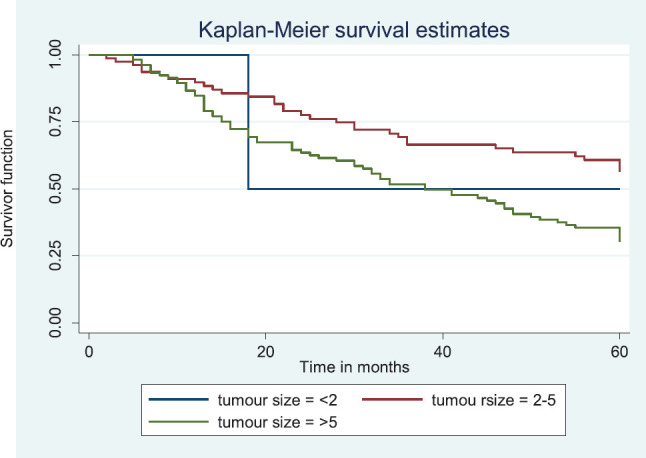
Kaplan–Meier curve by tumour size.

Figure 9 illustrates a Kaplan–Meier curve with a statistically significant difference in median survival times for patients with tumour size 2–5 cm and those with tumour size >5 cm. Log rank = 10.85, *p*-value = 0.0044. The median survival of the only four patients with tumour size <2 cm was lower than those with tumour size 2–5 cm.

**Figure 10.**
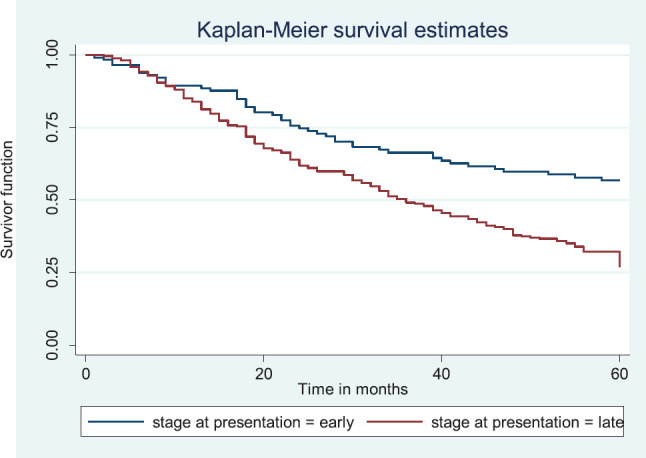
Kaplan–Meier curve by stage at presentation.

Figure 10 shows the Kaplan–Meier curve for stage at presentation with a statistically significant difference in median survival time for early and late stages, respectively. Log rank = 21.05, *p*-value≤0.0001.

**Figure 11.**
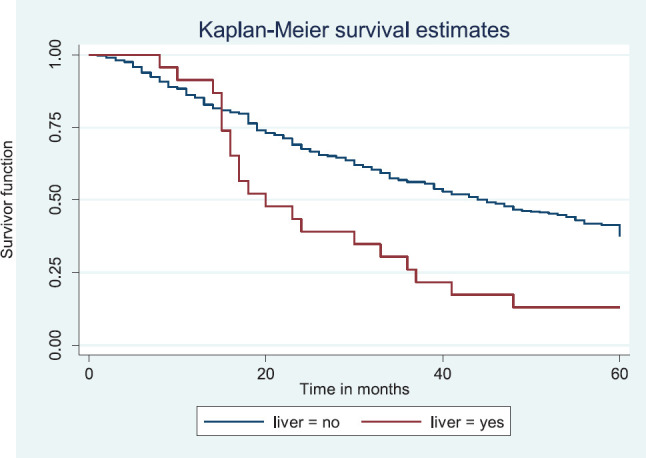
Kaplan–Meier curve by liver metastasis.

Figure 11 illustrates a Kaplan–Meier curve with a statistically significant difference in median survival times for patients with liver metastasis (20 months) and those without liver metastasis (45 months). Log rank = 10.05, *p*-value = 0.0015.

**Figure 12.**
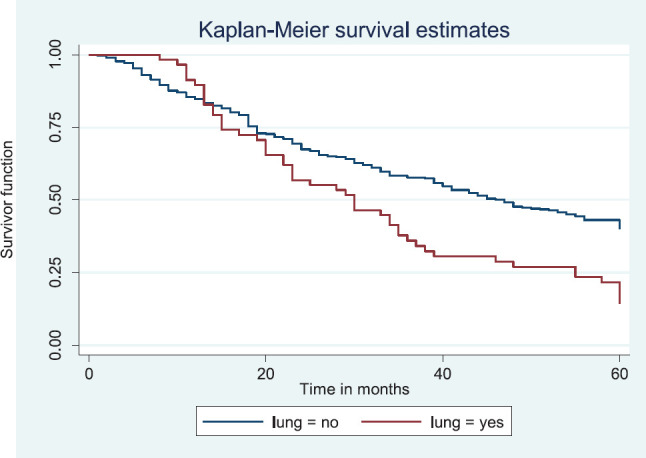
Kaplan–Meier curve by lung metastasis.

Figure 12 illustrates a Kaplan–Meier curve with a statistically significant difference in median survival times for patients with lung metastasis (30 months) and those without lung metastasis (47 months). Log rank = 12.09, *p*-value = 0.0005.

**Figure 13.**
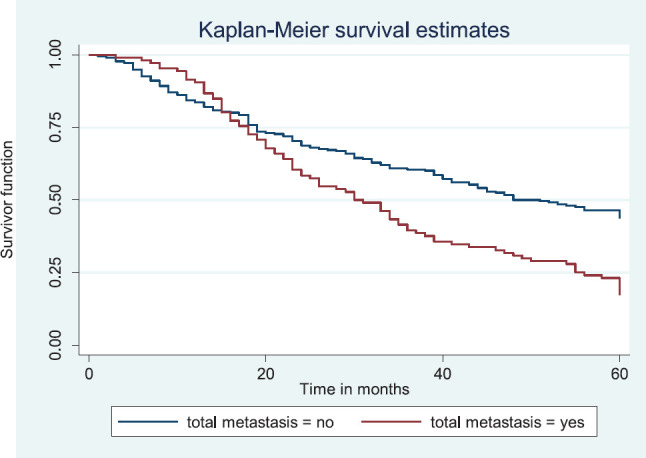
Kaplan–Meier curve by total metastasis.

Figure 13 reveals a statistically significant difference in median survival time of patients that had no metastasis (51 months) and those with metastasis (30 months). Log rank = 18.05. *p-*value≤0.0001.

**Figure 14.**
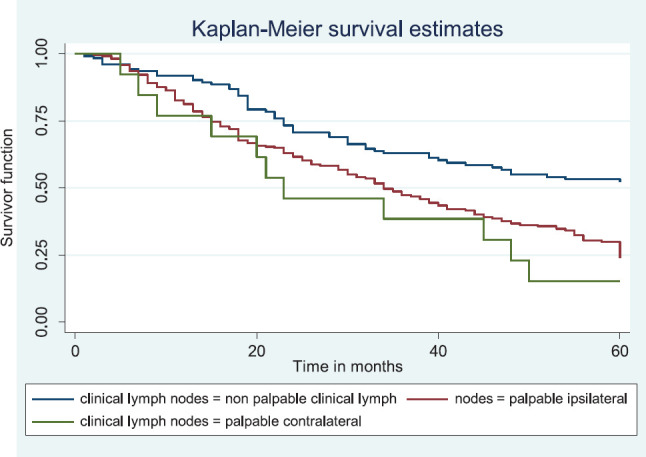
Kaplan–Meier curve by clinical axillary lymph node status.

Figure 14 shows the Kaplan–Meier curve for clinical axillary node metastasis with a statistically significant difference in median survival time for non-palpable, palpable ipsilateral and bilateral axillary nodes. This was statistically significant Log rank = 22.80, *p*-value≤0.001.

**Figure 15.**
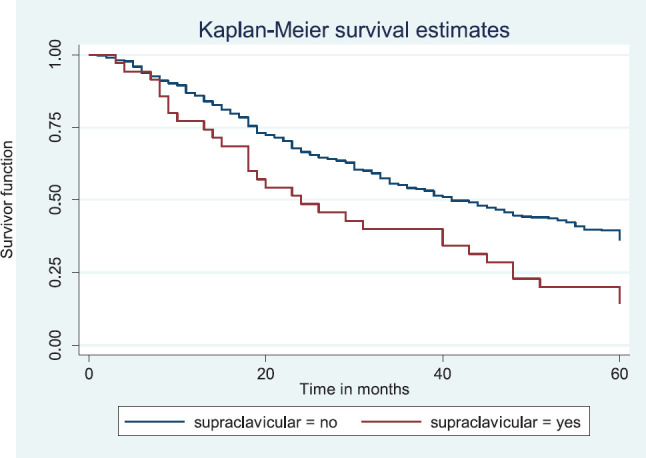
Kaplan–Meier curve by supraclavicular node metastasis.

Figure 15 shows the Kaplan–Meier curve for supraclavicular node metastasis with median survival time of 41 and 24 months for non-palpable and palpable supraclavicular nodes, respectively. This was statistically significant Log rank = 8.62, *p*-value≤0.0033.

### Comparative median survival times of patients among histological characteristics of respondents

There were no significant differences established in median survival time among histological characteristics of the patients. This is shown in Table 10.

### Comparative median survival times of patients among treatment modalities of respondents

Comparative median survival times of patients among treatment modalities are presented in Table 11. Patients with BCS had a statistically significant median survival time than those who had mastectomy, *p* = 0.0154. Similarly, patients with axillary clearance had a significantly lower median survival time compared to those who did not have axillary clearance, *p*≤0.001. Other variables did not have significant differences in their median survival time.

**Figure 16.**
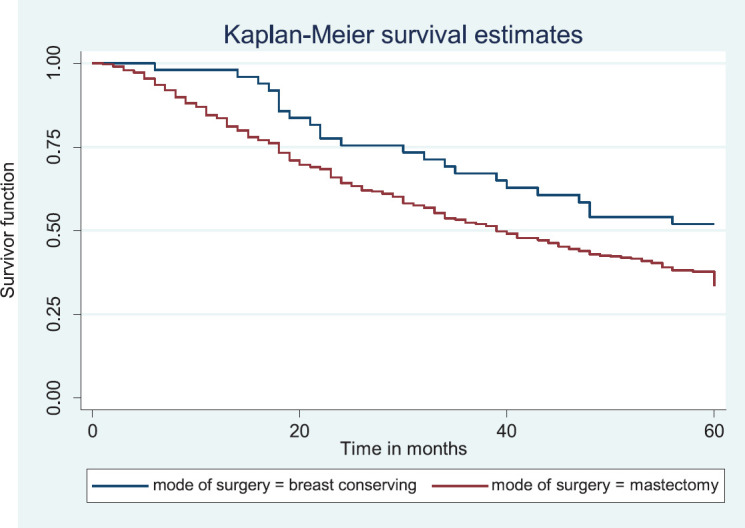
Kaplan–Meier curve by mode of surgery.

Figure 16 shows Kaplan–Meier curve which depict a statistically significant longer median survival time for those who had BCS against those who had mastectomy. Log rank = 5.87, *p-*value = 0.0154.

**Figure 17.**
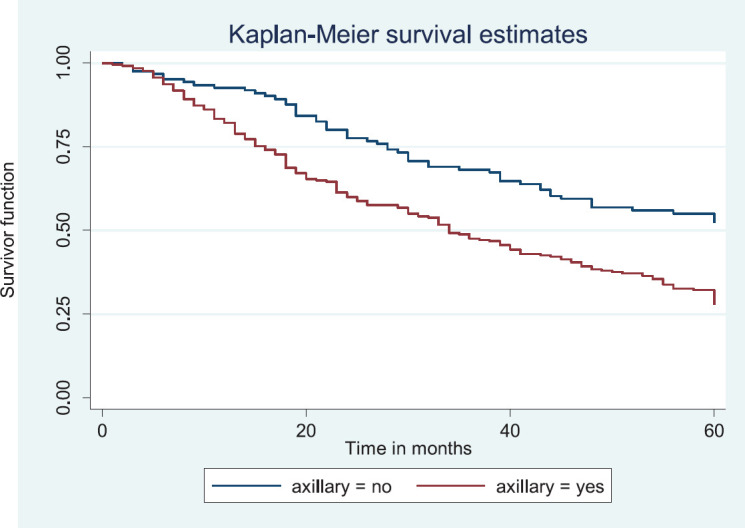
Kaplan–Meier curve by axillary clearance.

Figure 17 depicts the Kaplan–Meier curve for axillary clearance with longer median survival time for patients with axillary clearance than those without axillary clearance. This was statistically significant Log rank = 19.84, *p*-value≤0.001.

### Cox regression model for independent variables

Unadjusted and adjusted hazards ratios on variables are presented in Table 12. All variables had significant unadjusted hazard ratios except height. However, after adjusting for variables, only clinical tumour size (*p* = 0.028) remained significant.

## Discussion

The study found the mean age of breast cancer patients to be 47.6 years (SD 11.2) which is consistent with the findings of Adesunkanmi *et al* [18] in Ile-ife and studies in Ibadan by Dairo *et al* [9] and Ayandipo *et al* [19]. Similar findings were also demonstrated from north eastern part of Nigeria at the University of Maiduguri Teaching Hospital [20] and Federal Medical Centre, Gombe [21]. Retrospective study of breast cancer from north western Tanzania also revealed a mean age of 47.8 years which is in congruent to this study.

This is in sharp contrast to reports from Australia where the mean age at presentation was found to be 60.7 years [22] and in the US it was reported as 63 years for white Americans and 57 years for African Americans [7]. This further buttresses the fact that the average age at diagnosis in Africans is 10 to 15 years younger than that of the western world [23].

From the findings, 86.2% of the patients were married which corresponds to the 97.3% married breast cancer patients documented by the study at Ile-ife [18], the percentage distribution is also quite similar to those studied in the article titled ‘a case control study for breast cancer risk in Ibadan’ [24]. This can possibly be explained by the influence of culture and religion on social life of most Nigerians who prefer to stay in marriage and also see it as the norm for most of the African societies.

A large number of the patients were employed (85.4%), this is in contrast to a study in Ibadan which found a lower rate of employment at 44.4% [25], but other studies in Ibadan and Ile-ife found a similar high employment rate [18, 19].

The bulk of the patients (47.0%) belong to the low socioeconomic class. The educational status of the patients studied showed only 56.1% with formal education as opposed to other local studies which showed higher levels [24, 25]. Most of the patients seen were Yoruba—this is probably as a result of the location of the centre in South West Nigeria which is predominantly Yoruba speaking and since South Western Nigeria is known to be the region with the highest literacy level which may have influenced their presentation in the hospital for care. This is also seen in other breast cancer studies from the region [18, 24].

The majority of patients (78.8%) presented first after 6 months, and mean duration of illness at presentation was 18.4 months. Mean duration of illness of some studies is also late but shorter, for instance, mean of 12.2 months [26], 11.2 months [18] has been reported. Reasons for late presentation has been suggested to be fear of mastectomy [26, 27], ignorance, spiritual belief and herbal treatment [26]. In addition to these factors, the patients in these cohorts have a significant number of people with low level of education and also majority are from lower occupational classes which may also contribute to the delay in presentation as most patients pay out of pocket.

In this study, most patients present with unilateral breast cancer against only 4.2% with bilateral cancer. Left breast cancer was commoner (50.3%). These findings are consistent with that in Ibadan [9, 25], studies in Benin [28] and by Oguntola *et al* [29].

Most patients (98.9%) had a tumour ≥2 cm while 28.0% had tumours >5 cm. This is similar to what was reported by Ayandipo *et al* [19] in Ibadan, 93.3% of their patients had tumours ≥2 cm and 65.3% >5 cm in size. There are disparities in the size of the tumour at presentation in this study with that of the western world. In Poland, for example, the number of tumours detected with diameters ≤5 cm increased from 57% in 1984 to 81% in 2003 [30]. On the contrary, in Africa, only 52% of the patients have tumour size below 6 cm [31]. This can be explained by the long duration of illness and the advanced disease stage that most patients present with in this part of the world.

The majority of patients presented at late stages III and IV (62.9%), these finding was also reported by other local studies [6, 8, 12, 25, 26]. In addition to the reasons mentioned above for late presentation, higher number of patients in this study had a delayed presentation. All these factors (delay in presentation, fear of mastectomy, ignorance, spiritual belief, herbal treatment, low level of education and low socio-economic class) summed up together may account for higher stage at presentation seen in these patients.

About 28.0% of the patients developed metastasis, the frequency of distribution of metastasis to the liver, lung and bone is very similar to those reported in Nigerian literatures [25, 32]. The most common visceral metastasis in this study was lungs (15.3%), Anyanwu [33] also found lungs and vertebral tree to be the commonest site of metastasis. However, another Nigerian study puts the frequency of bone metastasis to be slightly higher than that of the lungs [9].

Palpable axillary node was detected in 61.9%, this is high when compared with what is documented in some studies to as low as 17.2% [25]. The frequency of supraclavicular node metastasis corresponds to what is obtainable in some local studies [25]. The relative higher rates of lymph node metastasis can partially be explained by the late presentation of the patients and probably the young age at presentation and aggressive tumour biology of breast cancer in Africans.

The main histology was invasive ductal carcinoma which constituted 89.2% of the cases. Most studies in the country have reported similar findings [9, 18–20, 25, 28].

Worldwide invasive ductal carcinoma is the commonest, but in some cases the proportion is as low as 50% [34]. Only a single case of carcinoma *in situ* was found, unlike developed countries which account for 10% of cases as a result of increased used of mammography for screening [18].

The composition of other rarer types of breast cancer seen was similar to the findings on rare breast cancers conducted in the same department [35]. Rare breast carcinomas are reported to be less than 10% in most literature [36].

In the majority of the patients (67.2%), the SBR grade is not known; however, poorly differentiated carcinoma constitutes 42.7% of the 124 patients who had the SBR grade documented. More African women have poorly differentiated carcinoma than their western counter parts. According to some studies, it constitutes about 45.1% of breast cancer cases in Nigeria [37] and 56.4% in Tanzania [31]. In contrast, most European women present with well or moderately differentiated carcinomas [38]. In some studies among African American women, poorly differentiated tumours were found to be associated with overexpression of cell cycle regulatory genes p53, p16 and cyclin E and also have high mitotic activity [39, 40]. The advanced stage at diagnosis is probably a consequence of rapid tumour growth from these genetic abnormalities.

Other pathological features such as immunohistochemistry, multi-focality, multi-centricity and lympho-vascular invasion were not documented for most patients as most referral hospitals done have facilities or expertise to do such investigations. In addition immunohistochemistry was not routinely done in Nigeria for the period studied.

More than half of the patients presented with late stage breast cancer and are not suitable candidates for BCS. In addition, the poor health care system in Africa affects the management of breast cancer as most patients are unable to undergo BCS due to lack of radiotherapy facilities near the hospital where they are being treated. Mastectomy rates in Africa are up to 80% [18], in contrast to just 30% in Europe [41]. The tumour biology and aggressiveness of breast cancers reported in Nigerians and hence in this cohort may have warranted high mastectomy rate. Most of the patients presented in stage III, therefore, required axillary clearance.

The overall 2- and 5-year survival rates of the breast cancer patients in our study were 56.4% and 37.6%, respectively, this is low when compared to studies in the western world despite selecting patients who had optimal treatment. The 2- and 5-year survival for early stage (I and II) was 68.7% and 58.3%, and late stage (III and IV) was 50.8% and 28.2%, respectively.

Some questions will arise from here as to what factors are responsible? One may infer that the early age onset coupled with aggressive disease, late presentation may account for these poor outcomes. Other possible explanations may be timing and sequencing of treatment, lack of immunohistochemistry data in most of the patients to adequately characterise and tailor treatment accordingly, and also availability and affordability of genuine chemotherapy drugs and other agents. For instance, a recent study of 251 Nigerian women with breast cancer found about half of them (47.4% ) are triple negative and only 15.9% are well differentiated [42]. This suggests our patients may have large number of triple negative breast cancer that may have affected the prognosis. However, further prospective and population based or multi-centre studies are needed to ascertain these assumptions.

The poor survival outcome in this study can also be attributed to low levels of education and socioeconomic class with most being poor and unable to pay for treatment or are ignorant or both [15]. The majority of patients have a long duration of symptoms, advanced stage, large tumour size and positive axillary node metastasis. As a result of few radiotherapy centres, the optimal timing of surgery, radiotherapy and chemotherapy may not be met.

Among the few studies available from Nigeria, most also report poor outcomes. Popoola *et al* [12] reported a 5-year overall survival of 25.6%, 45% for stage II, 15% for stage III and 5% for stage IV, the median survival time was 30 months. Kene *et al* [43] in a study of patients with breast cancer reported only 24.5% of patients with advanced breast cancer survive beyond 30 months. Anyanwu [33] documented a median survival time of 31.0 months. The values of the above studies are low compared to 41.0 months in our study.

The slightly better survival in this study may be attributed to the fact that the other studies in Nigeria mentioned did not make efforts to contact those lost to follow up as they used only patients that are still attending the hospital or whose status is available in the hospital records.

A study assessing the ‘Impact of axillary node-positivity and surgical resection margins on survival of women treated for breast cancer in Ibadan, Nigeria’ reported an overall 5-year survival rate of 65.3% [19] which is higher than in our study probably because it is prospective and are able to follow up the patients easily, but this is still low when compared to the Western World data.

Survival studies from southern Africa reported higher breast cancer survival than our study, for instance, in Uganda, the 5-year overall survival from Mulago hospital was reported as 56% [44], and in South Africa it was 80% in whites and 64% in blacks [11].

From Asia, in a hospital based study in Thailand, the 5-year survival rate of breast cancer for early stage was 60% and 27% late stage which is similar to what was obtained in this study [45].

The survival figures are much higher and keep increasing in the western world. From the EUROCARE-3 study, the 5-year overall breast cancer survival was about 80% for the Nordic region, and most central and southern Europe which is highest in Europe, while it is lowest at 60%–70% in Eastern Europe [46]. A key explanation in the differences in survival rate for these regions are likely to be more advanced stage of disease at diagnosis in the countries with lower survival rates [46], while in Eastern Europe, disparity in treatment may also plays a role [47]. According to the Canadian cancer statistics 2013, the 5-year breast cancer relative survival was 88% [48].

In the US, the American cancer society report on cancer facts and figures estimates breast cancer survival at 90.4% for white women and a lower value of 78.7% for black women [49]. The Uppsala-Orebro Breast Cancer Study Group randomised control study in the US which compared two groups of patients with stage I breast cancer with or without post-operative radiotherapy reported an 83.3% disease free survival at 10 years for early stage disease who had post-operative radiotherapy and also found that BCS plus radiotherapy resulted in an absolute reduction in local recurrence of 16% at 10 years compared with surgery alone [50].

Findings from a clinical trial conducted by the National Surgical Adjuvant Breast and Bowel Project put estimates of disease-free survival at 12 years for patients treated by lumpectomy and mastectomy with irradiation in early stage disease at 50% and 49%, respectively [51]. This also has a better outcome than the disease free survival in this study which was evaluated at lower intervals of 2 and 5 years. They also found that no significant differences in overall survival and disease free survival between the patients who underwent total mastectomy and those treated by lumpectomy with or without breast irradiation.

A study done in Brazil on disease-free survival in patients with non-metastatic breast cancer showed a disease free survival at 5 years of 72% [52]. This is higher than our cohort even though they considered only non-metastatic breast cancer at diagnosis. They also found lymph node involvement, use of hormone therapy and education level are independently associated with disease free survival. Their findings further buttressed the importance of early diagnosis for better disease free survival.

One of the challenges encountered in this study was the patient lost to follow up. About 25.1% of the patients were lost to follow. A study in Ibadan found that for breast cancer patients, the 5-year and 10-year discontinuation rates of follow up were 69.8% and 92.6%, respectively and discontinuers were more likely to be older than the age of 45 years, have metastasis, are anaemic and have late-stage disease [9]. The cohort in the above study was similar to ours since they were taken in the same hospital, so also findings of advanced disease. These may explain the high number lost to follow.

In this study, level of education, height, tumour unilaterality, clinical tumour size, stage at presentation, presence of metastases, clinical axillary lymph node metastasis, supraclavicular metastasis, mode of surgery and axillary clearance were found to have statistically significant association with survival.

Results from previous studies on education level and invasive breast cancer survival have been inconsistent [53, 54]. However, a study in Sweden showed that survival increased with increasing levels of education, university graduates were associated with the highest survival (lowest fatality hazard ratio), compared to those with education of less than 9 years [55]. Patients with no formal education are likely to belong to the low socioeconomic class, be ignorant about cancer and may easily accept spiritual believes and interventions and therefore will not seek medical attention until the disease is advanced leading to poorer outcome.

Several epidemiological studies have been done to investigate the effect of height on the risk for breast cancer, studies investigating effect of height on survival of cancer patients have also been brought to notice. In our study, there was significant increase in median survival time with increase in height. A Turkish retrospective reviewed of 393 women with breast cancer found a tendency for better survival in taller individuals but no significant variation between height groups [56]. A study on height and overall cancer risk and mortality from 310,000 UK biobank participants suggested that height increases the risk of being diagnosed with and dying from cancer [57].

In a retrospective cohort study of 1,907 Taiwanese women with breast cancer, it was found that the 15-year survival rates were better for unilateral breast cancer (68.37%) when compared with 62.63% and 26.42% for synchronous and metachronous bilateral breast cancer, respectively [58]. Differences were most apparent after 5 years of follow-up and also, the risk of death for overall bilateral breast cancer was 2.50-fold greater compared to unilateral breast cancer.

On the contrary, retrospective analysis of 403 patients with bilateral operable breast cancer treated at Memorial Sloan-Kettering Cancer Centre indicated no difference in overall survival between unilateral and bilateral breast cancer, they only found significant differences in the disease-free survival between these patients [59].

In a study in Ibadan clinic—pathologic parameters like large tumour size, invasive ductal carcinoma, grade, positive resection margin, number of harvested nodes, number of positive metastatic nodes and presence of loco-regional recurrence were associated with poor survival [19]. This further affirms the fact that the negative impact of traditional prognostic factors such as large tumour size, stage, palpable nodes and presence of metastasis on survival as reported in our study.

Data on 24,740 cases recorded in the Surveillance, Epidemiology, and End Results found survival rates of 45.5% for tumour diameters equal to or greater than 5 cm with positive axillary nodes to 96.3% for tumours less than 2 cm and with no involved nodes [60]. Tumour diameter and lymph node status were found to act as independent but additive prognostic indicators. As tumour size increased, survival decreased regardless of lymph node status; and as lymph node involvement increased, survival status also decreased regardless of the tumour size. The size of a tumour correlates with likelihood of metastasis, stage of the disease and sometimes the aggressiveness of the tumour. These may account for the low survival rate seen in those with larger tumour size in our study.

The relatively lower median survival of those with tumour size 2 cm compared to those with 2–5 cm in this study can be explained by the few numbers of those 2 cm (4 versus 79) and also the sometimes tumour size does not reflect the true stage of the disease considering the fact that a small tumour may have lymph node and/or distant metastases. The histological type and subtype may also account for the prognosis.

Axillary lymph node status is the most important factor affecting outcome [61]. The 5-year survival for patients with node-negative disease has been reported as 82.8% compared with 73% for 1–3 positive nodes, 45.7% for 4–12 positive nodes and 28.4% for ≥13 positive nodes [62]. There is direct relationship between axillary lymph node involvement and risk of distance metastasis, positive axillary status also indicates advanced disease and aggressive tumour both resulting in poor outcome.

Patients with positive supraclavicular metastasis were found to have lower survival than patients without supraclavicular lymph node metastasis. It has been shown that survival from the time of appearance of supraclavicular lymph node metastases at primary diagnosis or as a recurrence is not different from survival of patients presenting with a primary M_1_ stage or presenting with distant metastases during the course of disease [63]. The presence of supraclavicular nodes in breast cancer patients indicates advanced disease which has been demonstrated in this study to have poor outcome.

The median survival of individuals with metastatic breast cancer is 18–24 months, although the range in survival spans between a few months to many years and depends very much on the type of breast cancer the patient has [64]. Similarly, patients with metastatic disease have been shown to have poorer survival compared to other groups in our study.

Other studies have reported lower median survival time of 13 months (range 4–123+) from detection of lung metastases, with patients who had solitary lung metastasis surviving for a median of 11.5 months as compared to 10.5 months for patients with more than one pulmonary metastases [65]. A higher median survival time has also been reported after diagnosis and treatment of a solitary thoracic metastasis as 42 months and remission for up to 121 months has been reported [66].

It has been suggested that the use of chemotherapy, hormonal therapy or surgery may improve the overall survival of breast cancer patients with lung metastasis [67]. This may explain the relatively higher median survival time in patients with lung metastasis in this study as all have been treated with chemotherapy with or without hormonal therapy.

Liver metastases from breast cancer is also associated with a poor prognosis, median survival reported in literature without intervention is <6 months and patients may survive not more than 2 years when given chemo-hormonal therapy or supportive care alone [68]. However, a subgroup of these patients with no dissemination in other organs may benefit from other interventions.

A report of 17 women who underwent hepatic metastasectomy with curative intent for metastatic breast cancer had an actuarial 5-year survival rate of 22%, seven of the 17 patients were alive after follow-up for 12 years, the median survival was 63 months [68]. In another study of breast cancer patients with only liver metastasis who had resection with or without radiofrequency ablation, the overall 2- and 5-year survival rates were 86% and 61%, respectively, whereas the 2- and 5-year disease-free survival rates were 39% and 31%, respectively [69].

Therefore, from the reports in the aforementioned literatures also suggest that our patients with hepatic metastases also had a relatively higher median survival time as a result of the ‘optimal’ treatment received.

In this study, patients who did not have axillary clearance were also found to have improved outcome compared to those who had axillary clearance. Similarly, patients who had BCS had a statistically significant longer survival time than those who had mastectomy.

These outcomes should be interpreted with caution as all stages were considered together. Those patients with early stages are likely to have BCS and no axillary clearance, and in this study patients with early stage were found to have better survival. Therefore, these patients are most likely those with early stage breast cancer.

On the contrary, the positive impact of lymph node dissection when indicated has been suggested in literature [70, 71] and also the outcome of BCS and mastectomy in early breast cancer is similar [72].

The poor survival outcome in this study in comparison to what is obtainable in western literature can be attributed to late presentation with majority in stage III, requiring either axillary nodes clearance and/or mastectomy, hence the poor outcome.

## Conclusion

A large number of the patients in our study presented at a young age, late with advanced stage disease which results in poor survival outcome. Level of education, height, tumour unilaterality, clinical tumour size, stage at presentation, presence of metastases, clinical axillary lymph node metastasis, supraclavicular metastasis, mode of surgery and axillary clearance were found to have statistically significant association with survival.

## Limitations of the study

The study is retrospective. We had a relatively low sample size because more than 50% of the patients treated during the period did not have complete data or were excluded based on the study criteria. The study was a single hospital based, so generalisation of the result of the study needs to be made with caution. There are also a significant number of patients that were lost to follow up. There are no funds to visit patients lost to follow-up outside Ibadan to determine their disease status.

Most patients had no immunohistochemistry as characterisation of the breast cancer subtypes may go a long way in determining treatment type or shaping the outcome of our patients and it will also allow comparison of outcome of such subset of patients with similar patients in another study, in order to reach a conclusion on the disparity in outcomes. So there is also lack of documentation for other pathologic features such as multi-centricity, multi-focality and lympho-vascular invasion that can be used to assess the outcome. The optimal timing and sequencing for surgery, chemotherapy and radiotherapy may not have been made as most patients are referred from far places due to few radiotherapy centres in the country.

Despite all the limitations, the study has highlighted important data on the outcome of breast cancer patients which can generate questions for further research. This knowledge can also be used to in setting policies toward breast cancer treatment.

## Funding

We received no funding for the writing of this paper.

## Competing interests

All authors declare that they have no competing interests.

## Figures and Tables

**Table 1.** Socio-demographic characteristics of respondents.

Variable	Frequency	Percentage
**Age** **Mean (SD**) 20–29 30–39 40–49 50–59 60–69 70–79 80+ Total	47.6 (11.1) 7 84 143 79 49 14 2 378	22.0–87.0 1.9 22.2 37.8 20.9 13.0 3.7 0.5 100.0
**Sex** Female Male Total	372 6 378	98.4 1.6 100.0
**Marital status** Single Married Widowed Divorced Total	17 326 32 3 378	4.5 86.2 8.5 0.8 100.0
**Level of education** Non-formal Formal Total	166 212 378	43.9 56.1 100.0
**Employment status** Employed Unemployed Total	323 55 379	85.4 14.6 100.0
**Occupation class** Class I Class II Class III Class IV Class V Class VII Clergy Retired Total	23 89 60 9 137 41 4 15 378	6.1 23.5 15.9 2.4 36.2 10.8 1.1 4.0 100.0
**Ethnic group** Yoruba Igbo Hausa Others Total	178 114 2 84 378	47.1 30.2 0.5 22.2 100.0

**Table 2.** Physical characteristics of respondents.

Variable	Frequency	Percentage
**Height (m)** <1.5 1.5–1.7 >1.7 Not documented Total	10 265 17 86 378	2.6 70.1 4.5 22.8 100.0
**Weight (kg)** <60 60+ Not documented Total	49 239 90 378	13.0 63.2 23.8 100.0
**BMI** Normal Overweight Obese	85 118 85	22.5 31.2 22.5

**Table 3.** Tumour characteristics and metastases.

Variable	Frequency	Percentage
**Tumour site** Right Left Bilateral Total	172 190 16 378	45.5 50.3 4.2 100.0
**Clinical tumour size (cm)** <2 2–5 >5 Not documented Total	4 79 106 189 378	1.1 20.9 28.0 50.0 100.0
**Bone metastasis** No Yes Total	342 36 378	90.5 9.5 100.0
**Liver metastasis** No Yes Total	355 23 378	93.9 6.1 100.0
**Lung metastasis** No Yes Total	320 58 378	84.7 15.3 100.0
**Spine** No Yes Total	351 27 378	92.9 7.1 100.0
**Total metastasis** No Yes Total	272 106 378	72.0 28.0 100.0
**Clinical axillary lymph nodes metastasis** Non-palpable Palpable ipsilateral Palpable contra lateral Not documented Total	125 221 13 19 378	33.1 58.5 3.4 5.0 100.0
**Supraclavicular lymph node** No Yes Not documented Total	318 35 25 378	84.1 9.3 6.6 100.0

**Table 4.** Histology.

Variable	Frequency	Percentage
**Histology ** Invasive ductal carcinoma Invasive lobular carcinoma Others Total	337 25 16 378	89.2 6.6 4.2 100
SBR grade Well Moderate Poorly Missing Total	25 46 53 254 378	6.6 12.2 14.0 67.2 100

**Table 5.** Treatment modalities.

Variable	Frequency	Percentage
**Chemotherapy** Neoadjuvant Adjuvant Total	40 338 378	10.6 89.4 100
**Nature of chemotherapy** CAF CMF Taxanes AC Others Total	160 100 2 91 25 378	42.3 26.5 0.5 24.1 6.6 100
**Radiotherapy** Neo-adjuvant Adjuvant Total	5 373 378	1.3 98.7 100
**Mode of surgery** BCS Mastectomy Total	50 328 378	13.2 86.8 100
**Axillary clearance** No Yes Total	124 254 378	32.8 67.2 100
**Hormonal treatment** No Yes Total	138 240 378	36.5 63.5 100

CMF: Cyclophosphamide, Methotrexate, 5-FU; AC: Adriamycin, Cyclophosphamide

**Table 6.** Five-year survival rates by selected variables.

Variable, Frequency(*N*)	2-year survival rate (%)	5-year survival rate (%)
**Age (years), *N* = 378** <40 >40	53.3 57.1	35.6 38.0
**Stage at presentation, *N* = 353** Early (0–II) Late (III–IV)	68.7 50.8	58.3 28.2
**Stage of presentation, *N* = 353** Stage 0 Stage I Stage II Stage III Stage IV	100.0 80.0 67.7 51.4 37.9	100.0 66.7 57.6 27.9 13.8
**Duration of illness, *N* = 352** <12 months >12 months	57.4 55.2	39.0 36.7

**Table 7.** Comparative median survival times of patients among selected socio-demographic variables for all the 378 patients.

Variable	Median survival time (months) 95%CI	Log rank test	*p* value
**Age (years)** <40 40+	41.0 (25.0–54.0) 43.0 (34.0–52.0)	0.60	0.4283
**Sex** Female Male	41.0 (36.0–48.0) 26.0 (7.0–)	0.00	0.9572
**Marital status** Married Others	43.0 (36.0–49.0) 30.0 (24.0–60.0)	0.58	0.4449
**Level of education** Non-formal Formal	33.0 (28.0–43.0) 48.0 (39.0–60.0)	4.66	0.0309
**Class** High Middle Low Unclassified	54.0 (38.0–) 39.0 (25.0–51.0) 39.0 (30.0–47.0) 39.0 (9.0–)	3.79	0.2850
**Ethnic group** Yoruba Igbo Others	44.0 (36.0–53.0) 44.0 (31.0–60.0) 34.0 (24.0–52.0)	1.95	0.3769

**Table 8.** Comparative median survival times of patients among selected variables.

Variable, Frequency(*N*)	Median survival time (months) 95%CI	Log rank test	*p* value
**Height (m), *N* = 292** <1.5 1.5–1.7 >1.7	17.0 (5.0–32.0) 44.0 (35.0–56.0) -	8.37	0.0153
**Weight (kg), *N* = 288** <60 60+	45.0 (22.0–) 45.0 (36.0–56.0)	0.00	0.991
**BMI, *N* = 288** Normal Overweight Obese	45.0 (34.0–60.0) 48.0 (31.0–60) 34.0 (25.0–54.0)	1.03	0.5972
**Duration of illness, *N* = 252** <6 months 6–12 months >12 months	60.0 (25.0–) 36.0 (26.0–47.0) 44.0 (34.0–55.0)	2.99	0.2246

**Table 9.** Comparative median survival times of patients by tumour characteristics and metastasis.

Variable, frequency(*N*)	Median survival time (months) 95%CI	Log rank test	*p* value
**Tumour site, *N* = 378** Right Left Bilateral	47.0 (35.0–60.0) 41.0 (33.0–48.0) 17.0 (13.0–50.0)	6.42	0.0403
**Clinical tumour size (cm), *N* = 182** <2 2–5 >5	18.0 (18.0–) - 38.0 (30.0–48.0)	10.85	0.0044
**Stage of presentation, *N* = 353** Early stage Late stage	- 36.0 (31.0–43.0	21.05	<0.001
**Bone metastasis, *N* = 378** No Yes	43.0 (35.0–48.0) 39.0 (26.0–60.0)	0.72	0.3974
**Liver metastasis, *N* = 378** No Yes	45.0 (39.0–53.0) 20.0 (16.0–33.0)	10.05	0.0015
**Lung metastasis, *N* = 378** No Yes	47.0 (40.0–55.0) 30.0 (22.0–36.0)	12.09	0.0005
**Spine, *N* = 378** No Yes	41.0 (34.0–48.0) 43.0 (21.0–60.0)	0.68	0.4083
**Total metastasis, *N* = 378** No Yes	51.0 (41.0–60.0) 30.0 (24.0–36.0)	18.05	<0.001
**Clinical axillary lymph nodes metastasis, *N* = 359** Non-palpable Palpable ipsilateral Palpable contra lateral	-(43.0–) 34.0 (29.0–41.0) 23.0 (9.0–48.0)	22.80	<0.001
**Supraclavicular lymph node, *N* = 353** No Yes	41.0 (34.0–49.0) 24.0 (18.0–40.0)	8.62	0.0033

**Table 10.** Comparative median survival times of patients among histological characteristics of respondents.

Variable	Median survival time (months) 95%CI	Log rank test	*p* value
**Histology ** Invasive ductal carcinoma Invasive lobular carcinoma Others	41.0 (35.0–48.0) 33.0 (15.0–60.0) 52.0 (22.0–	2.02	0.3640
**SBR grade** Well Moderate Poorly	39.0 (16.0–) 60.0 (30.0–) 35.0 (23.0–60.0)	0.3	0.6932

**Table 11.** Comparative median survival times of patients among treatment modalities of respondents.

Variable	Median survival time (months) 95%CI	Log rank test	*p* value
**Chemotherapy** Neoadjuvant Adjuvant	41.0 (18.0–) 41.0 (35.0–51.0)	0.12	0.7330
**Radiotherapy** Neoadjuvant Adjuvant	53.0 (16.0–) 41.0 (35.0-48.0)	0.05	0.8221
**Mode of surgery** BCS Mastectomy	-(39.0) 39.0 (33.0–46.0)	5.87	0.0154
**Axillary clearance** No Yes	- (45.0-) 34.0 (30.0-41.0)	19.84	<0.001
**Hormonal treatment** **No** Yes	39.0 (30.0–52.0) 44.0 (36.0–54.0)	0.80	0.3704

**Table 12.** Cox regression model for independent variables.

Variable	Unadjusted		Adjusted	
	HR (95%CI)	*p* value	HR (95%CI)	*p* value
**Education** Non-formal (ref) Formal	0.76 (0.58–0.98)	0.033	0.74 (0.46–1.20)	0.228
**Height (m)** <1.7 (ref) >1.7	0.59 (0.31–1.12)	0.107	0.38 (0.09–1.59)	0.185
**Tumour site** Unilateral (ref) Bilateral	1.96 (1.14–3.37)	0.015	0.97 (0.34–2.76)	0.956
**Clinical tumour size (cm)** <5 (ref) >5	1.94 (1.30–2.94)	0.001	2.19 (1.09–4.43)	0.028
**Stage of presentation** Early stage (ref) Late stage	2.07 (1.50–2.85)	<0.001	0.68 (0.28–1.66)	0.397
Liver metastasis	2.06 (1.30–3.27)	0.002	1.29 (0.46–3.46)	0.643
Lung metastasis	1.73 (1.26–2.37)	0.001	1.63 (0.77–3.45)	0.198
Total metastasis	1.76 (1.35–2.30)	<0.001	1.09 (0.55–2.16)	0.801
**Clinical axillary lymph nodes metastasis** Non-palpable (ref) Palpable	1.79 (1.40–2.29)	<0.001	1.67 (0.90–3.09)	0.103
Supraclavicular lymph node	1.76 (1.20–2.58)	0.004	1.80 (0.69–4.70)	0.231
**Mode of surgery** BCS (ref) Mastectomy	1.68 (1.09–2.59)	0.018	0.67 (0.26–1.74)	0.410
Axillary clearance	1.94 (1.44–2.63)	<0.001	1.92 (0.87–4.23)	0.105

HR: hazard ratio
